# Association of Midkine and Pleiotrophin Gene Polymorphisms With Systemic Lupus Erythematosus Susceptibility in Chinese Han Population

**DOI:** 10.3389/fimmu.2020.00110

**Published:** 2020-02-21

**Authors:** Peng Wang, Yan-Mei Mao, Chan-Na Zhao, Jie-Bing Wang, Xiao-Mei Li, Dong-Qing Ye, Hai-Feng Pan

**Affiliations:** ^1^Center for Genetic Epidemiology and Genomics, School of Public Health, Medical College of Soochow University, Suzhou, China; ^2^Department of Epidemiology and Biostatistics, School of Public Health, Anhui Medical University, Hefei, China; ^3^Anhui Province Key Laboratory of Major Autoimmune Diseases, Hefei, China; ^4^Department of Rheumatology and Immunology, Anhui Provincial Hospital, Hefei, China

**Keywords:** midkine, pleiotrophin, single nucleotide polymorphisms, systemic lupus erythematosus, autoimmune diseases

## Abstract

In a previous study, we have reported an increased plasma midkine (MK) and pleiotrophin (PTN) concentrations in patients with systemic lupus erythematosus (SLE) and the increase in MK and PTN associated with inflammatory cytokines interleukin (IL)-17 level and some clinical manifestations, suggesting the underlying association of MK and PTN with SLE. This study was conducted to investigate the association between common single-nucleotide polymorphisms (SNPs) in the *MK* and *PTN* gene and SLE susceptibility. A total of 989 subjects (496 SLE patients and 493 healthy controls) were included and genotyped for three *MK* SNPs and seven *PTN* SNPs in using improved multiple ligase detection reaction (iMLDR). Results have demonstrated no significant differences for genotype and allele frequencies in all 10 SNPs between SLE patients and healthy controls. Case-only analysis in SLE revealed that, in *MK* gene, the genotype frequency of AA/AG (rs35324223) was significantly lower in patients with photosensitivity than those without; the allele frequency of A/G (rs20542) was significantly higher in patients without serositis. In *PTN* gene, the A/G allele frequency (rs322236), C/T allele frequency, and TT/CT genotype frequency (rs6970141) showed significantly increased results in patients with immunological disorder compared to those without. Furthermore, no significant differences in plasma MK and PTN concentrations with its SNPs genotypes were found. *MK* and *PTN* SNPs showed no associations with SLE genetic susceptibility, but it may be associated with the course of this disease; further studies are needed to focus on the mechanism of *MK* and *PTN* genes in the pathogenesis of SLE.

## Introduction

Systemic lupus erythematosus (SLE) is a chronic and severe systemic autoimmune disease involving multiple organs/tissues, characterized by the production of antinuclear autoantibodies, complement and interferon activation, and tissue destruction (1–3). SLE could occur at any age, particularly during childbearing years and predominantly affecting women (at a 9:1 ratio) (2). Up to now, no single cause for SLE has been identified. Epidemiological evidence, together with recent linkage and association studies, have demonstrated that the interactions between predisposing genetic factors and environmental components are hypothesized to contribute to the pathogenesis of SLE (4–8).

Midkine (MK), representing as the founding member of heparin-binding growth factor family, was initially identified as the product of the *MK* gene, which is 1.5 kb in size and located on chromosome 11q11.2 (9, 10). MK has a similar structure and shares 50% homology in amino acid sequence with pleiotrophin (PTN) (11). MK and PTN are the only cytokines that constitute the *PTN*/*MK* developmental gene family and show a biological activity including the promotion of growth, cell migration, tissue morphogenesis, and chemokine expression in numerous target cell types (12, 13). During the past few years, increasing evidences suggested an essential role of MK and PTN in carcinomas and acute and chronic inflammatory diseases (14–16). Studies have demonstrated that MK and PTN are involved in several types of carcinomas; the overexpression of MK or PTN correlated with a poor prognosis for patients with neuroblastomas, urinary bladder carcinomas, and papillary thyroid cancer (17–20). Furthermore, studies have shown an association of intronic polymorphism of *MK* gene with human sporadic colorectal and gastric cancers (21, 22). In postmenopausal women, *PTN* gene promoter −1227C>T (rs321198) polymorphism contributed to the genetic background of osteoporosis (23).

In rheumatoid arthritis (RA), a study has found an increased serum MK level in RA patients and its correlation with disease activity score (DAS) 28-erythrocyte sedimentation rate (ESR) and rheumatoid factors (RFs) titer (24). Our previous study has revealed that, as compared to healthy controls, the levels of plasma MK and PTN are elevated in SLE and also associated with interleukin (IL)-17 levels and some clinical manifestations, including rash and anti-Sjögren's-syndrome-related antigen A (anti-SSA) (25). These findings suggest a potential role of MK and PTN involved in certain types of autoimmune diseases.

Although study has unveiled the genetic association of *MK* and *PTN* in human cancer, the association between common single-nucleotide polymorphisms (SNPs) in the *MK* and *PTN* genes and SLE susceptibility has not yet been elucidated. Therefore, the present study was undertaken to comprehensively evaluate the role of common genetic variations in *MK* and *PTN* gene with SLE susceptibility in a Chinese population.

## Materials and Methods

### Study Population

This case–control genotyping study consisted of 989 study subjects. Four hundred ninety-six SLE patients were recruited from the Department of Rheumatology and Immunology at Anhui Provincial Hospital, the First Affiliated Hospital of Anhui Medical University. All patients were diagnosed based on a strict medical history and immunological autoantibody screening results, as well as with at least four 1997 American College of Rheumatology (ACR) revised criteria for SLE; in addition, an attending rheumatologist (Xiao-Mei Li) was also invited to confirm the diagnosis of SLE. The patients were then classified based on the 1997 revised ACR classification criteria (26, 27). Individual disease activity was quantified using the SLE Disease Activity Index 2000 (SLEDAI-2K) score (28, 29). The disease activity of SLE was stratified according to SLEDAI-2K score, of which a SLEDAI-2K score of ≥10 or <10 was defined as more active or less active, respectively (30). Four hundred ninety-three geographically and ethnically matched healthy controls were enrolled in the current study. All of the healthy controls did not have any inflammatory/autoimmune diseases history. Demographics, clinical manifestations, and laboratory findings were collected from hospital medical records and then reviewed by experienced physicians.

The Ethical Committee of Anhui Medical University (Hefei, Anhui, China) approved this study. The present study was conducted in accordance with the Declaration of Helsinki. All subjects, both cases and controls, provided informed consent to participate in this study.

### SNP Screening and Genotyping

We used Ensembl Gene Browser 37 (http://grch37.ensembl.org/index.html) to acquire the detailed genetic and location information of human *MK* and *PTN* genes (31), and downloaded the linkage pedigree file (PED) and marker information file in Chinese Han population (CHB) of Beijing. Then, candidate tag SNPs selection was applied by utilizing the Haploview 4.2 software (Broad Institute, Cambridge, MA, United States), with the linkage disequilibrium *r*^2^ ≥ 0.80 and minor allele frequency (MAF) ≥ 5%. In total, 41 candidate tag SNPs (2 MK tag SNPs, 39 PTN tag SNPs) were chosen based on prior criteria. The online bioinformatics tools (https://snpinfo.niehs.nih.gov/snpinfo/snpfunc.html) were implemented to predict the function of 41 tag SNPs (32), including potentially deleterious functional impact at the splicing, transcriptional, translational, and post-translational level, as shown in Table S1.

In addition, relevant literatures reporting SNPs regarding *MK* and *PTN* gene polymorphisms were also reviewed. Finally, in our study cohort, we included three tag SNPs (rs116869512, rs20542, and rs35324223) in *MK* gene and seven tag SNPs (rs161335, rs321198, rs322236, rs3959914, rs6970141, rs919581, and rs322297) in *PTN* gene for further genotyping. The detailed information regarding the location of the SNPs within each gene locus is displayed in Table S2.

### Plasma MK and PTN Detections

Intravenous blood samples (5 ml) of SLE patients were collected; the plasma sample was extracted and then frozen at −80°C in a refrigerator until assayed. Determinations of MK and PTN concentrations in plasma were performed using enzyme-linked immunosorbent assay (ELISA) kits (purchased from Anhui Xinle Biotechnology Co. LTD; expressed as pg/ml).

### Statistical Analysis

Comparisons of genotype and allele frequencies of SNPs between cases and controls were undertaken using chi-square or Fisher's exact test. Differences in plasma MK and PTN levels between different genotypes were compared using non-parametric test. The unconditional logistic regression model was used to estimate the associations between genotypes and SLE susceptibility. Three models were considered for statistical analysis, including additive, dominant, and recessive models. Statistical analysis was performed with the use of the Statistical Package for the Social Sciences (SPSS) statistical software, version 23.0 (SPSS Inc., Chicago, IL, United States).

Hardy–Weinberg equilibrium (HWE) and haplotype analyses were implemented using the SHEsis software (http://analysis.bio-x.cn/myAnalysis.php) (33). All statistical tests with two-tailed *P* < 0.05 values were considered statistically significant. The Bonferroni correction was used for multiple testing to reduce the chances of obtaining false-positive results.

## Results

### Basic Characteristics of Study Subjects

This study recruited 496 SLE patients and 493 healthy controls. In SLE patients, there were 438 female and 58 male with an average age of 37.58 ± 11.44 years; the disease duration was 5.78 ± 5.59 years, and the average SLEDAI-2K scores were 11.40 ± 9.07. In addition, the body mass index (BMI) for patients was 21.83 ± 3.17. As for healthy controls, there were 434 female and 59 male with an average age of 38.45 ± 11.32 years. The age and gender distribution showed no significant differences between cases and controls. Demographic characteristics and clinical features of study subjects are summarized in Table 1. The major clinical features of SLE patients comprised immunological disorder (73.2%), hematological disorder (68.1%), malar rash (54.6%), arthritis (49.4%), photosensitivity (31.1%), and renal disorder (37.1%) (Table 1). In healthy controls, the presence of observed genotype frequency distributions of all included tag SNPs were not significantly different from the HWE at the 5% level.

**Table 1:** Demographic characteristics and clinical features of patients with SLE and control subjects.

**Parameters**	**Patients with SLE (*n* = 496)**	**Healthy controls (*n* = 493)**
**Demographic characteristics**		
Age (years)	37.58 ± 11.44	38.45 ± 11.32
Female, *n* (%)	438 (88.3)	434 (88.0)
Male, *n* (%)	58 (11.7)	59 (12.0)
Disease duration (year)	5.78 ± 5.59	NA
BMI (kg/m^2^)	21.83 ± 3.17	NA
SLEDAI-2K	11.40 ± 9.07	NA
**Disease manifestations**		NA
Malar rash, *n* (%)	271 (54.6)	NA
Discoid rash, *n* (%)	94 (19.0)	NA
Photosensitivity, *n* (%)	194 (31.1)	NA
Oral ulcers, *n* (%)	119 (24.0)	NA
Arthritis, *n* (%)	245 (49.4)	NA
Serositis, *n* (%)	45 (9.1)	NA
Renal disorder, *n* (%)	184 (37.1)	NA
Neurological disorder, *n* (%)	21 (4.2)	NA
Hematological disorder, *n* (%)	338 (68.1)	NA
Immunological disorder, *n* (%)	363 (73.2)	NA

*BMI, body mass index; n, number; SLE, systemic lupus erythematosus; SLEDAI-2K, Systemic Lupus Erythematosus Disease Activity Index 2000*.

### Association of MK and PTN Gene Polymorphisms With Susceptibility to SLE

The genotype and allele frequencies of *MK* and *PTN* genes in SLE and healthy controls are shown in Tables 2, 3. In *MK* gene, there was no significant difference in genotype and allele distributions of three tag SNPs in SLE patients compared to healthy controls (Table 2). When analyzing seven tag SNPs in *PTN* gene, we did not find any significant differences in genotype and allele frequencies between SLE patients and healthy controls (Table 3).

**Table 2:** Genotype frequency of *MK* SNPs in SLE patients and healthy controls.

**SNPs**	**Analyzed model**		**SLE**	**Control**	*P-*value^*^
rs116869512	Genotypes	CC	448	452	0.999
		CA	45	41	0.999
		AA	3	0	
	Additive model	CC	448	452	0.999
		AA	3	0	
rs20542	Genotypes	GG	424	420	0.725
		GA	67	70	0.494
		AA	5	3	
	Additive model	GG	424	420	0.725
		AA	5	3	
rs35324223	Genotypes	AA	434	421	0.405
		AG	52	65	0.596
		GG	2	4	
	Additive model	AA	434	421	0.405
		GG	2	4	

*SLE, systemic lupus erythematosus; SNPs, single nucleotide polymorphisms; MK, midkine*.

* *The P-values are not corrected for multiple testing, Bonferroni corrected P = 0.005*.

**Table 3:** Genotype frequency of *PTN* SNPs in SLE patients and healthy controls.

**SNPs**	**Analyzed model**		**SLE**	**Control**	*P-*value^*^
rs161335	Genotypes	CC	164	182	0.383
		CT	255	239	0.990
		TT	77	72	
	Additive model	CC	164	182	0.383
		TT	77	72	
rs321198	Genotypes	CC	161	163	0.901
		CT	253	245	0.704
		TT	82	85	
	Additive model	CC	161	163	0.901
		TT	82	85	
rs322236	Genotypes	AA	453	437	0.999
		GA	38	56	0.999
		GG	5	0	
	Additive model	AA	453	437	0.062
		GG	5	0	
rs3959914	Genotypes	CC	155	155	0.862
		CT	245	244	0.871
		TT	95	92	
	Additive model	CC	155	155	0.862
		TT	95	92	
rs6970141	Genotypes	TT	450	452	0.998
		CT	45	39	0.569
		CC	1	1	
	Additive model	TT	450	452	0.998
		CC	1	1	
rs919581	Genotypes	AA	338	344	0.819
		GA	143	135	0.977
		GG	15	14	
	Additive model	AA	338	344	0.819
		GG	15	14	
rs322297	Genotypes	TT	493	493	0.999
		TG	3	0	

*SLE, systemic lupus erythematosus; SNPs, single nucleotide polymorphisms; PTN, pleiotrophin*.

* *The P-values are not corrected for multiple testing, Bonferroni corrected P = 0.005*.

### Association of MK and PTN Gene Polymorphisms With Clinical Manifestations in SLE

To unveil the possible genetic associations in *MK* and *PTN* gene polymorphisms with clinical manifestations, case-only analysis was applied. In *MK* gene, as shown in Table 4, the frequency of AA/AG genotype (rs35324223) was significantly lower in patients with photosensitivity than those without (*P* = 0.012). The allele frequency of A/G (rs20542) was significantly higher in patients without serositis (*P* = 0.042). In *PTN* gene, the A/G allele frequency (rs322236), C/T allele frequency (rs6970141), and TT/CT genotype frequency appeared significantly increased risks in patients with immunological disorder compared to those without (*P* = 0.020, *P* = 0.027, *P* = 0.035, respectively) (Table 5). However, there were no significant associations for other tag SNPs in *MK* and *PTN* genes with clinical disease manifestations.

**Table 4:** The positive findings on association of clinical characteristics with genotype and allele frequencies in *MK*.

**SNPs**	**Allele (M/m)**	**Clinical features**	**Group**	**Genotypes (*****n*****)**			**OR (95% CI)**	***P-*value**	**Alleles (*****n*****)**		**OR (95% CI)**	***P-*value**
				**MM**	**Mm**	**mm**			**M**	**m**		
rs35324223	A/G	Photosensitivity	Positive	175	13	2	0.493 (0.256, 0.951)	**0.012**	363	17	0.669 (0.373, 1.200)	0.205
			Negative	259	39	0			557	39		
rs20542	A/G	Serositis	Positive	1	10	34	0.698 (0.480, 1.016)	0.090	12	78	0.505 (0.261, 0.975)	**0.042**
			Negative	4	57	390			65	837		

*Values in bold show significance*.

*SNPs, single nucleotide polymorphisms; OR, odds ratio; MK, midkine*.

**Table 5:** The positive findings on association of clinical characteristics with genotype and allele frequencies in *PTN*.

**SNPs**	**Allele (M/m)**	**Clinical features**	**Group**	**Genotypes (*****n*****)**			**OR (95% CI)**	***P-*value**	**Alleles (*****n*****)**		**OR (95% CI)**	***P-*value**
				**MM**	**Mm**	**mm**			**M**	**M**		
rs322236	A/G	Immunological disorder	Positive	337	24	2	1.297 (0.663, 2.537)	0.068	698	28	2.027 (1.121, 3.663)	**0.020**
			Negative	116	14	3			246	20		
rs6970141	C/T	Immunological disorder	Positive	1	39	323	2.621 (1.085, 6.334)	**0.035**	41	685	2.594 (1.088, 6.182)	**0.027**
			Negative	0	6	127			6	260		

*Values in bold show significance*.

*SNPs, single nucleotide polymorphisms; OR, odds ratio; PTN, pleiotrophin*.

### Association of Plasma MK and PTN Levels With Its Genotypes

The results indicated that, in patients with SLE, there were no significant differences in plasma MK and PTN concentrations with its tag SNPs genotypes (Tables 6, 7).

**Table 6:** Association of plasma MK levels with genotype in *MK* gene.

**SNPs**	**Genotypes**	**Number**	**Plasma MK levels (pg/ml)**	***P*-value**
			**M (P_**25**_, P_**75**_)**	
rs116869512	CA	9	1651.18 (1199.71, 2684.59)	0.250
	CC	75	2085.79 (1627.88, 2610.80)	
rs20542	GA	13	1944.15 (1618.05, 2621.05)	0.771
	AA	1	2008.56	
	GG	70	2074.96 (1611.07, 2618.65)	
rs35324223	AG	7	1639.78 (1608.53, 1657.19)	0.271
	AA	76	2074.96 (1613.76, 2648.43)	
	GG	1	2610.80	

*SNPs, single nucleotide polymorphisms; M, median; MK, midkine*.

**Table 7:** Association of plasma PTN levels with genotype in *PTN* gene.

**SNPs**	**Genotypes**	**Number**	**Plasma PTN levels (pg/ml)**	***P*-value**
			**M (P_**25**_, P_**75**_)**	
rs161335	CT	47	762.52 (698.84, 943.86)	0.303
	CC	24	704.67 (666.77, 820.88)	
	TT	13	766.78 (673.57, 846.91)	
rs321198	TC	42	742.85 (682.69, 912.54)	0.802
	CC	31	763.50 (691.60, 914.56)	
	TT	11	723.56 (666.06, 887.05)	
rs322236	GA	5	761.56 (689.55,856.13)	0.605
	AA	79	738.07 (681.00, 902.10)	
rs322297	TT	84	742.85 (681.56, 899.32)	–
rs3959914	CT	42	754.60 (687.50, 919.94)	0.946
	CC	24	718.32 (652.19, 847.07)	
	TT	18	795.18 (668.69, 912.54)	
rs6970141	CT	6	846.45 (676.32, 906.04)	0.824
	CC	1	651.23	
	TT	77	738.07 (683.77, 908.33)	
rs919581	GA	25	761.56 (691.93, 939.20)	0.909
	AA	55	732.46 (674.15, 886.98)	
	GG	4	744.21 (696.38, 898.53)	

*SNPs, single nucleotide polymorphisms; M, median; PTN, pleiotrophin*.

### Haplotype Analyses

We have constructed four main haplotypes (AAA, CAA, CGA, and CGG) for *MK* gene and eight main haplotypes (CCATCTA, CCATTTG, CTATCTA, CTATTTA, CTATTTG, TCATCTA, TCATTTA, and TCATTTG) for *PTN* gene using SHEsis software. The results revealed that *MK* and *PTN* genes haplotypes were not associated with SLE susceptibility (Tables 8, 9).

**Table 8:** Haplotype analysis of three SNPs in *MK* gene in SLE patients and healthy controls.

**Haplotype**	**SLE [*n* (%)]**	**Controls [*n* (%)]**	**χ^**2**^**	***P*-value**	**OR (95% CI)**
rs116869512–rs20542–rs35324223					
AAA	45.86 (4.7)	39.23 (4.0)	0.588	0.443	1.186 (0.767, 1.833)
CAA	27.99 (2.9)	35.04 (3.6)	0.766	0.381	0.798 (0.482, 1.323)
CGA	842.09 (86.5)	830.73 (84.9)	1.222	0.269	1.156 (0.894, 1.495)
CGG	52.85 (5.4)	70.22 (7.2)	2.496	0.114	0.744 (0.514, 1.075)

*Total χ^2^ = 3.811, P = 0.282. All the haplotypes with a frequency < 0.03 were ignored in the analysis*.

*SLE, systemic lupus erythematosus; SNPs, single nucleotide polymorphisms; MK, midkine; OR, odds ratio*.

**Table 9:** Haplotype analysis of seven SNPs in *PTN* gene in SLE patients and healthy controls.

**Haplotype**	**SLE [*n* (%)]**	**Control [*n* (%)]**	**χ^**2**^**	***P-*value**	**OR (95% CI)**
rs161335–rs321198–rs322236–rs322297–rs3959914–rs6970141–rs919581					
CCATCTA	81.52 (8.2)	97.66 (9.9)	1.867	0.171	0.806 (0.591, 1.099)
CCATTTG	51.11 (5.4)	48.59 (4.9)	0.154	0.694	1.084 (0.726, 1.618)
CTATCTA	126.09 (12.7)	128.94 (13.1)	0.095	0.758	0.959 (0.735, 1.251)
CTATTTA	202.14 (20.4)	211.45 (21.5)	0.460	0.497	0.926 (0.743, 1.155)
CTATTTG	49.37 (5.0)	47.41 (4.8)	0.019	0.890	1.029 (0.683, 1.551)
TCATCTA	294.27 (29.7)	279.44 (28.5)	0.314	0.575	1.059 (0.867, 1.293)
TCATTTA	31.93 (3.2)	21.83 (2.2)	1.823	0.177	1.460 (0.840, 2.538)
TCATTTG	44.04 (4.4)	35.48 (3.6)	0.848	0.357	1.236 (0.786, 1.944)

*Total χ^2^ = 5.061, P = 0.653. All the haplotypes with a frequency < 0.03 were ignored in the analysis*.

*SLE, systemic lupus erythematosus; SNPs, single nucleotide polymorphisms; PTN, pleiotrophin; OR, odds ratio*.

## Discussion

MK and PTN comprise a two-member family of heparin-binding cytokines. Previous studies have demonstrated that MK and PTN can be highly expressed in various human cancers and play a key role in the promotion of cancer cell survival, proliferation, and angiogenesis, contributing to tumor growth. *MK* gene promoter contains a putative nuclear factor-kappa B (NF-kB)-responsive element that can drive the induction of MK (34). In the early of 2000, Ahmed et al. has examined the entire coding region, four regions of the promoter and eight sets of intron-based and promoter region primers; they found that, in the *MK* promoter region, mainly a G/T polymorphism (G to T transition) at the 62 nd base of intron 3, there was a higher G/T genotype frequency in colorectal cancers, suggesting that this G/T genotype might be a risk factor contributing to the carcinoma in the colon and rectum (35, 36). However, Lai et al. reported that the genetic variation of *MK* gene (rs20542) was not associated with sporadic gastric cancers (21).

For PTN, interferon (IFN)-g could positively regulate and promote the expression of PTN via an IFN-g-responsive promoter element (37). Besides, hyaluronan (HA) has been shown to inhibit the toll-like receptor (TLR) 4 activity to downregulate PTN level in a Th1-type autoimmune disease model, implicating *PTN* as a TLR4-responsive gene (38). Mencej-Bedrac et al. has addressed the relationship of *PTN* gene with osteoporosis; they found that the *PTN* gene rs321198 polymorphisms and its CT haplotype were associated with genetic susceptibility of osteoporosis in postmenopausal women (23).

It has also been disclosed that MK plays critical roles in several types of inflammatory diseases. MK was expressed by macrophage- and fibroblast-like cells of the synovial membrane. Enhanced MK levels on both synovial fluid and sera were present in RA patients, and the increase in MK level positively correlated with RF (24). In RA patients with active inflammatory synovitis, the inflamed tissues showed an overexpression of MK, while a non-inflamed tissue had no MK expression at all (39).

We previously reported elevated plasma MK and PTN concentrations in SLE compared with healthy controls, and the increase in MK and PTN levels correlated with inflammatory cytokine IL-17 and some clinical features/parameters, including rash and anti-SSA antibody, suggesting that an aberrant expression of MK and PTN might be involved in the progress of inflammation and the course of SLE (25).

In the present study, we investigated the association of *MK* and *PTN* gene SNPs and its susceptibility to SLE in a Chinese population. A total of 10 tag SNPs of *MK* and *PTN* genes were genotyped and analyzed; however, we did not find any significant associations of 10 tag SNPs with SLE susceptibility. In addition, we also performed a case-only analysis to determine the relationship of 10 tag SNPs with the clinical features of SLE; our results showed that the SNPs in *MK* and *PTN* genes associated with some SLE clinical manifestations. In the *MK* gene, the frequency of AA/AG genotypes (rs35324223) was decreased in SLE patients with skin photosensitivity, suggesting that the lower AA/AG genotype of rs35324223 might correlate with decreased skin photosensitivity risk and play as a protective factor for the occurrence of skin photosensitivity. Moreover, we identified a decreased A/G (rs20542) allele frequency in patients with serositis; the decreased A/G allele frequency appeared to be protective against serositis in SLE. In the *PTN* gene, we found that two SNPs (rs322236 and rs6970141) associated with an increased risk for immunological disorder in SLE; the A allele of rs322236, T allele, and TT/CT genotype of rs6970141 were the risk alleles of immunological disorder, contributing to the disease clinical features. To observe the possible associations of *MK* and *PTN* SNPs with their plasma protein levels, we analyze the differences in plasma MK and PTN levels in different genotypes of each tag SNPs, but there were no correlations between plasma levels of MK and PTN levels and its tag SNPs. At last, the haplotype analysis in our study revealed no significant differences in *MK* and *PTN* gene haplotypes. Taken together, although there were no associations of the 10 tag SNPs in *MK* and *PTN* genes with SLE susceptibility, we have revealed that there are some SNPs that interact together in modulating the risk toward clinical manifestation of skin photosensitivity, serositis, and immunological disorder in SLE.

To the best of our knowledge, this is the first study to investigate the association of *MK* and *PTN* gene SNPs with SLE susceptibility. Nevertheless, several shortcomings of the present study should be acknowledged. First, only part of the patients had plasma MK and PTN measurements, which may cause the potential bias. Second, only common variants in *MK* and *PTN* genes were examined; it may reduce the effect of these SNPs in their association analyses. Furthermore, in the present study, only Chinese Han population was included; it may restrict the generalizability of our results.

## Conclusions

Overall, results from the present study revealed that *MK* and *PTN* gene polymorphisms have no associations with SLE genetic susceptibility; however, we found associations of some tag SNPs with specific SLE clinical manifestations, suggesting that *MK* and *PTN* genes associated with the course of SLE. However, further studies with a larger study sample size and more ethnic lines covering the entire gene variability of both *MK* and *PTN* genes are still required to confirm our results.

## Ethics Statement

This study was approved by the Ethical Committee of Anhui Medical University (Hefei, Anhui, China). All the study subjects provided informed consent to participate in this study.

## Consent for Publication

We have obtained consent to publish from the participant (or legal parent or guardian for children) to report individual patient data.

## Author Contributions

PW, H-FP, and D-QY participated in the design of this study, analyzed the results, and finalized the manuscript. J-BW and Y-MM carried out all the ELISA analyses. C-NZ and X-ML contributed sera of their SLE patients and assisted in analyzing the clinical data of these patients. All authors read and approved the final manuscript.

### Conflict of Interest

The authors declare that the research was conducted in the absence of any commercial or financial relationships that could be construed as a potential conflict of interest.
