# Supplementary material for: Association of Midkine and Pleiotrophin Gene Polymorphisms With Systemic Lupus Erythematosus Susceptibility in Chinese Han Population
Source: Front Immunol. 2020 Feb 21;11:110. doi: 10.3389/fimmu.2020.00110 (PMC7046794; doi:10.3389/fimmu.2020.00110)
Supplement: Supplementary file 1 [file Table_1.DOC]

**Table S1** Characteristics of the 41 Tag SNPs

| rs_num | Chromosome | Position | Allele | Gene Name | CHB_  Hapmap | Prediction | | | | | | | | | | | | |
| --- | --- | --- | --- | --- | --- | --- | --- | --- | --- | --- | --- | --- | --- | --- | --- | --- | --- | --- |
|  |  |  |  |  |  | TFBS | Splicing (site) | Splicing (ESE or ESS) | Splicing (abolish domain) | miRNA (miRanda) | miRNA (Sanger) | nsSNP | Stop Codon | Polyphen | SNPs3D (svm profile) | SNPs3D (svm structure) | RegPotential | Conservation |
| rs20542 | 11 | 46360225 | A/G | MDK | -- | -- | -- | Y | -- | -- | -- | -- | -- | -- | -- | -- | 0.582909 | 0.002 |
| rs35324223 | 11 | 46359428 | A/G | MDK | -- | Y | -- | -- | -- | -- | -- | -- | -- | -- | -- | -- | 0.537255 | 0 |
| rs10256915 | 7 | 136563763 | A/G | PTN | 0.685 | -- | -- | -- | -- | -- | -- | -- | -- | -- | -- | -- | 0.047504 | 0 |
| rs10268599 | 7 | 136661747 | G/T | PTN | 0.667 | -- | -- | -- | -- | -- | -- | -- | -- | -- | -- | -- | 0 | 0.03 |
| rs10488607 | 7 | 136570708 | C/G | PTN | 0.9 | -- | -- | -- | -- | -- | -- | -- | -- | -- | -- | -- | 0 | 0.019 |
| rs10954576 | 7 | 136564309 | A/G | PTN | 0.675 | -- | -- | -- | -- | -- | -- | -- | -- | -- | -- | -- | 0 | 0.002 |
| rs10954577 | 7 | 136565355 | A/G | PTN | 0.393 | -- | -- | -- | -- | -- | -- | -- | -- | -- | -- | -- | 0 | 0 |
| rs1162024 | 7 | 136669878 | A/G | PTN | -- | -- | -- | -- | -- | -- | -- | -- | -- | -- | -- | -- | NA | 0 |
| rs11764598 | 7 | 136602480 | C/T | PTN | 0.838 | -- | -- | -- | -- | -- | -- | -- | -- | -- | -- | -- | NA | 0 |
| rs12668246 | 7 | 136654984 | A/G | PTN | 0.75 | -- | -- | -- | -- | -- | -- | -- | -- | -- | -- | -- | 0.03727 | 0 |
| rs12674065 | 7 | 136660897 | A/T | PTN | 0.932 | -- | -- | -- | -- | -- | -- | -- | -- | -- | -- | -- | 0.032078 | 0.222 |
| rs13228711 | 7 | 136668869 | A/T | PTN | 0.9 | -- | -- | -- | -- | -- | -- | -- | -- | -- | -- | -- | 0 | 0 |
| rs13242861 | 7 | 136572301 | C/T | PTN | -- | -- | -- | -- | -- | -- | -- | -- | -- | -- | -- | -- | 0 | 0.008 |
| rs13245564 | 7 | 136639844 | A/G | PTN | 0.72 | -- | -- | -- | -- | -- | -- | -- | -- | -- | -- | -- | 0 | 0 |
| rs13245911 | 7 | 136640146 | A/G | PTN | 0.554 | -- | -- | -- | -- | -- | -- | -- | -- | -- | -- | -- | 0.055811 | 0 |
| rs161335 | 7 | 136680885 | C/T | PTN | 0.633 | Y | -- | -- | -- | -- | -- | -- | -- | -- | -- | -- | 0.024955 | 0 |
| rs16874924 | 7 | 136595564 | C/G | PTN | 0.767 | -- | -- | -- | -- | -- | -- | -- | -- | -- | -- | -- | 0 | 0.006 |
| rs17168999 | 7 | 136570581 | C/T | PTN | 0.69 | -- | -- | -- | -- | -- | -- | -- | -- | -- | -- | -- | 0 | 0 |
| rs17169022 | 7 | 136611130 | C/G | PTN | 0.798 | -- | -- | -- | -- | -- | -- | -- | -- | -- | -- | -- | 0 | 0.001 |
| rs17169031 | 7 | 136617447 | C/T | PTN | 0.922 | -- | -- | -- | -- | -- | -- | -- | -- | -- | -- | -- | 0 | 0 |
| rs17169050 | 7 | 136626218 | A/G | PTN | 0.81 | -- | -- | -- | -- | -- | -- | -- | -- | -- | -- | -- | 0 | 0.001 |
| rs1839780 | 7 | 136563965 | C/T | PTN | 0.952 | -- | -- | -- | -- | -- | -- | -- | -- | -- | -- | -- | 0 | 0.001 |
| rs2290268 | 7 | 136661129 | C/A | PTN | 0.467 | -- | -- | -- | -- | -- | -- | -- | -- | -- | -- | -- | 0.074167 | 0 |
| rs321198 | 7 | 136680378 | C/T | PTN | 0.567 | Y | -- | -- | -- | -- | -- | -- | -- | -- | -- | -- | 0.08886 | 0 |
| rs322236 | 7 | 136605439 | A/G | PTN | 0.964 | -- | -- | -- | -- | -- | -- | -- | -- | -- | -- | -- | 0 | 0 |
| rs322238 | 7 | 136606672 | C/G | PTN | 0.689 | -- | -- | -- | -- | -- | -- | -- | -- | -- | -- | -- | 0 | 0.363 |
| rs322240 | 7 | 136607530 | A/C | PTN | 0.679 | -- | -- | -- | -- | -- | -- | -- | -- | -- | -- | -- | 0 | 0 |
| rs322294 | 7 | 136571810 | C/T | PTN | 0.661 | -- | -- | -- | -- | -- | -- | -- | -- | -- | -- | -- | 0.015976 | 0 |
| rs322297 | 7 | 136585999 | T/G | PTN | 1 | -- | -- | -- | -- | -- | -- | -- | -- | -- | -- | -- | 0 | 0.003 |
| rs322309 | 7 | 136600022 | T/C | PTN | -- | -- | -- | -- | -- | -- | -- | -- | -- | -- | -- | -- | NA | 0 |
| rs322335 | 7 | 136653944 | T/C | PTN | 0.756 | -- | -- | -- | -- | -- | -- | -- | -- | -- | -- | -- | 0 | 0 |
| rs322343 | 7 | 136645666 | G/C | PTN | -- | -- | -- | -- | -- | -- | -- | -- | -- | -- | -- | -- | NA | 0 |
| rs322349 | 7 | 136639981 | G/A | PTN | -- | -- | -- | -- | -- | -- | -- | -- | -- | -- | -- | -- | 0 | 0 |
| rs3959914 | 7 | 136621641 | C/T | PTN | 0.452 | -- | -- | -- | -- | -- | -- | -- | -- | -- | -- | -- | 0 | 0 |
| rs55862641 | 7 | 136604223 | C/T | PTN | -- | -- | -- | -- | -- | -- | -- | -- | -- | -- | -- | -- | NA | NA |
| rs61600011 | 7 | 136633188 | A/T | PTN | -- | -- | -- | -- | -- | -- | -- | -- | -- | -- | -- | -- | NA | NA |
| rs6970141 | 7 | 136562354 | C/T | PTN | 0.952 | -- | -- | -- | -- | -- | -- | -- | -- | -- | -- | -- | 0 | 0.001 |
| rs6977749 | 7 | 136636044 | C/T | PTN | 0.744 | -- | -- | -- | -- | -- | -- | -- | -- | -- | -- | -- | 0.080857 | 0 |
| rs7797731 | 7 | 136593247 | A/G | PTN | 0.864 | -- | -- | -- | -- | -- | -- | -- | -- | -- | -- | -- | 0 | 0.013 |
| rs833388 | 7 | 136603209 | T/C | PTN | -- | -- | -- | -- | -- | -- | -- | -- | -- | -- | -- | -- | NA | 0 |
| rs919581 | 7 | 136630714 | A/G | PTN | 0.807 | -- | -- | -- | -- | -- | -- | -- | -- | -- | -- | -- | 0 | 0 |

CHB, Han Chinese in Beijing, China
