# Supplementary material for: Association of Midkine and Pleiotrophin Gene Polymorphisms With Systemic Lupus Erythematosus Susceptibility in Chinese Han Population
Source: Front Immunol. 2020 Feb 21;11:110. doi: 10.3389/fimmu.2020.00110 (PMC7046794; doi:10.3389/fimmu.2020.00110)
Supplement: Supplementary file 2 [file Table_2.DOCX]

| SNPs | Position | Alleles | Gene | Gene Region | Variation Type |
| --- | --- | --- | --- | --- | --- |
| rs116869512 | chr11:46383776 (GRCh38.p12) | C>A | Midkine | 3’UTR | SNV |
| rs20542 | chr11:46382099 (GRCh38.p12) | G>A | Midkine | Exon2 | SNV |
| rs35324223 | chr11:46381302 (GRCh38.p12) | A>G | Midkine | Intron1 | SNV |
| rs161335 | chr7:137345599 (GRCh38.p12) | C>T | Pleiotrophin | 2KB Upstream Variant | SNV |
| rs321198 | chr7:137345092 (GRCh38.p12) | T>C | Pleiotrophin | 2KB Upstream Variant | SNV |
| rs322236 | chr7:137270152 (GRCh38.p12) | A>G | Pleiotrophin | Intron1 | SNV |
| rs3959914 | chr7:137286354 (GRCh38.p12) | C>T | Pleiotrophin | Intron1 | SNV |
| rs6970141 | chr7:137227067 (GRCh38.p12) | T>C | Pleiotrophin | 500B Downstream Variant | SNV |
| rs919581 | chr7:137295427 (GRCh38.p12) | A>G | Pleiotrophin | Intron1 | SNV |
| rs322297 | chr7:137250712 (GRCh38.p12) | T>G | Pleiotrophin | Intron4 | SNV |

**Table S2 Detailed information of the 10 included tag SNPs**

SNPs: single nucleotide polymorphisms; SNV: single nucleotide variation; UTR: untranslated regions
